# Detection and Recognition of Tilted Characters on Railroad Wagon Wheelsets Based on Deep Learning

**DOI:** 10.3390/s23187716

**Published:** 2023-09-07

**Authors:** Fengxia Xu, Zhenyang Xu, Zhongda Lu, Chuanshui Peng, Shiwei Yan

**Affiliations:** 1 School of Mechanical and Electrical Engineering, Qiqihar University, Qiqihar 161000, China; xufengxia_hit@163.com (F.X.); xuzhenyang329@163.com (Z.X.); 2 Qiqihar Sida Railway Equipment Co., Ltd., Qiqihar 161006, China; 13303628298@163.com (C.P.); yanshiwei3@163.com (S.Y.)

**Keywords:** deep learning, tilted characters, detection and recognition, Faster RCNN, CRNN

## Abstract

The quality of railroad wheelsets is an important guarantee for the safe operation of wagons, and mastering the production information of wheelsets plays a vital role in vehicle scheduling and railroad transportation safety. However, when using objection detection methods to detect the production information of wheelsets, there are situations that affect detection such as character tilting and unfixed position. Therefore, this paper proposes a deep learning-based method for accurately detecting and recognizing tilted character information on railroad wagon wheelsets. It covers three parts. Firstly, we construct a tilted character detection network based on Faster RCNN for generating a wheelset’s character candidate regions. Secondly, we design a tilted character correction network to classify and correct the orientation of flipped characters. Finally, a character recognition network is constructed based on convolutional recurrent neural network (CRNN) to realize the task of recognizing a wheelset’s characters. The result shows that the method can quickly and effectively detect and identify the information of tilted characters on wheelsets in images.

## 1. Introduction

Railroad freight transportation is a kind of freight transportation mode with long transportation distances, large cargo volume, and fast operation speed; it plays a vital role in the transportation network of each country [1,2,3]. Railroad wagon wheelsets are essential to operating safety, and they are designed, manufactured, and used according to strict standards and specifications. Railroad wagon wheelsets cast many character marks in production, including information on specifications, models, and others [4]. This information helps monitor vehicle mileage and axle wear. It is also significant to rationalize vehicle dispatch and ensure truck operation safety [5].

Traditional manual character recognition methods are time consuming and inefficient. Wheelset character recognition using machine vision is more effective than conventional recognition methods; however, it is more sensitive to light changes, image deformation, angle changes, and other disturbing factors. Therefore, studying an automatic wheelset character recognition method is of great practical significance.

With the development of deep learning techniques, object detection and recognition have significantly improved. The literature [6] proposes a connectionist text proposal network (CTPN) network, combined with the long short-term memory (LSTM) network based on Faster RCNN, to construct a horizontal detection. The algorithm sets the text as a fixed-width picture and predicts the whole bounding box through complex post-processing. The study of [7] makes the TextBoxes network after improving on SSD; the method regresses the entire bounding box and optimizes the anchor box size and convolution kernel size, thus adapting to the target text. The above deep learning methods recognize the character information in the horizontal direction, achieving good results and providing new ideas for the character detection methods.

Character recognition technology in the horizontal direction is becoming increasingly mature, and character recognition technology with a tilt angle has gradually become a concern. In [8], they improve the CTPN text detection network and change the side-refinement detection box to determine the scaling mechanism. A natural scene text recognition method built by combining YOLOv3 and the CRNN network was proposed [9]; it uses the text orientation detection algorithm to determine the tilted text lines fed into the CRNN network for text recognition. The study of [10] improves the CTPN multidirectional text detection algorithm and uses the CRNN algorithm for text recognition.

In this paper, an efficient and lightweight method based on deep learning is proposed for detecting and recognizing tilted characters of railroad wagons. The region of interest is first extracted using a detection network for a given railroad wagon wheel pair image. Afterward, the feature information is fed into the correction network to classify and correct orientation. Finally, it is fed into the recognition network to recognize the character information. The main contributions include the following:

1. We construct a tilted character detection network based on Faster RCNN. To increase detection speed, adding an architecture combining the feature pyramid network (FPN) and path aggregation network (PAN) performs feature extraction, using a two-branch structure to process horizontal and oriented boxes. Using the distance-IOU (DIOU) in the region proposal network (RPN) enhances the accuracy of the candidate box.
2. A lightweight network is designed to perform character orientation classification and correction; the MobileNet_ECA network, a combination of MobileNetV2 and efficient channel attention (ECA), is constructed as an individual learner with ensemble learning, and the Bagging algorithm is used to train it on different datasets to improve the accuracy of the network.
3. A network that recognizes characters has been constructed based on CRNN, using MobileNetV2_ECA as a feature extraction network to improve network speed and accuracy. The extracted features are fed into the bidirectional long short-term memory network (BLSTM) to remove the sequence information further. Finally, the connectionist temporal classification (CTC) is used to output the corresponding sequence of characters to realize the recognition of wheel–pair characters.

The main structure of this paper is as follows: Section 2 describes the traditional and deep learning methods used for tilted character recognition; Section 3 describes the proposed method, including the tilted character detection network, tilted character correction network, and character recognition network; Section 4 demonstrates the model’s performance through experiments; Section 5 gives conclusions based on the contents of this paper.

## 2. Related Work

Many algorithms have been proposed for recognizing tilted characters in complex environments, which can be categorized into traditional and deep learning methods.

### 2.1. Conventional Methods

Traditional methods mainly include manual inspection combined with computer technology for character recognition, including template matching, feature-based engineering, and other methods [11,12].

The template-matching method indexes a given image against a source image in the dataset. It uses a scoring method to determine the best location of the image in the dataset. However, this method takes a long time to match and cannot accurately handle large-scale datasets. Feature engineering is the process of transforming raw data into features that better express the nature of the problem, allowing the application of these features to predictive models to improve the accuracy of model predictions for invisible data. The method requires a lot of data processing work, while the complexity of the model makes the process inefficient when dealing with generalization work [13,14].

The traditional recognition object is an image with apparent color and contrast. In comparison, image acquisition may be more complex in the industrial production environment, such as poor lighting conditions, physical damage, and other interference. Traditional image processing recognition methods face these problems with low recognition accuracy and poor applicability.

### 2.2. Deep Learning Methods

Most deep learning methods for detecting characters rely on convolutional neural networks. The basic neural network architectures used in this paper are Faster RCNN [15], MobileNetV2 [16], and CRNN [17] networks.

For target detection, a range of region-CNN (RCNN) [18] methods perform well, and one of the most representative frameworks is Faster RCNN. This network architecture extracts features from the input image through a feature extraction network. It inputs the feature information into the RPN to roughly generate a candidate bounding box containing the target. After that, Fast RCNN further refines the bounding box by combining RPN’s suggestions and feature information from the feature extraction network; the region of interest (ROI) pooling helps Faster RCNN accelerate target detection with global features. The excellent performance and detection capabilities exhibited by the high-precision model Faster RCNN have led to an increasing number of detection networks being improved based on it [19,20,21].

Tuning the structure of deep neural networks to obtain the optimal balance between accuracy and efficiency is an essential area of research. MobileNetV1 [22] proposes the idea of depth-separable convolution, which separates the ordinary 3 × 3 convolution into 3 × 3 depthwise convolution and 3 × 3 pointwise convolution. The method significantly improves the computational efficiency. MobileNetV2 introduces a linear bottleneck and inverted residual structures for building more efficient layer structures. The inverted residual design is first convolved for dimension uplift, then through depth, and finally for dimension down the lift, extracting more feature information. Using the linear bottlenecks avoids the loss of feature information when going from a high-dimensional space to a low-dimensional space.

Many networks have improved recognition accuracy for single characters but are less efficient and are gradually being replaced by text line recognition methods, CRNN is the most widely used [23,24]. The CRNN architecture is divided into three parts: the image first passes through the convolutional layer for feature information extraction, and the extracted feature information is converted into a sequence output to the recurrent layer; the recurrent layer uses a bidirectional recurrent neural network to predict the feature sequences output from the convolutional layer, learns each feature vector in the sequence and outputs the expected label distributions. Using the CTC loss, the transcription layer maps the predictions of the label distributions from the recurrent layer into the lexicon’s sequence labels. It ultimately outputs a sequence of the corresponding text labels.

## 3. The Proposed Approach

**Figure 1:**
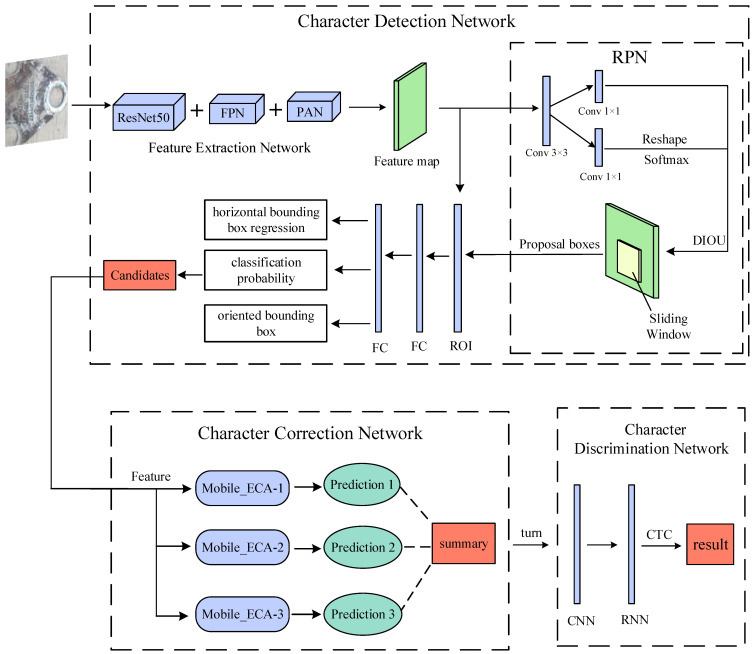
Overall system network architecture.

We propose a method for detecting and recognizing tilted characters on railroad wagon wheelsets. It is divided into three processes: a character detection network for feature image extraction and candidate box generation, a character correction network that classifies and corrects the orientation of the tilted character, and a character recognition network for detecting and recognizing character information. The framework of the proposed method is shown in Figure 1.

In Figure 1, the feature extraction network extracts the feature maps from the input images. Part of the feature maps are fed into the RPN architecture to generate proposal boxes, and the other part is fed into the ROI together with the proposal boxes output from the RPN, which outputs three vectors after passing through the two fully connected (FC) layers and finally outputs the candidates. After the image is fed to the character correction network, three predictions are generated by three MobileNetV2_ECA networks. The three prediction results are processed using the weighted voting method to output the final prediction summary and correct the image. The adjusted image is fed to the character recognition network, which outputs the information of the characters after the convolutional neural network (CNN), recurrent neural network (RNN), and CTC loss, where CNN uses MobileNetV2 and RNN uses BLSTM.

### 3.1. Tilted Character Detection Network

Depending on the type of railroad wagon, different character information is embossed on the wheelset. These characters have no absolute position and are prone to angular deflection. In addition, inconsistencies in the size of the characters can also affect the detection results. To solve these problems and enhance the network detection ability, this paper adopts Faster RCNN for improvement. The specific structure is as follows.

#### 3.1.1. Feature Extraction Network

The main task of the feature extraction network is to extract feature information from the input image to improve the feature extraction ability of the network for the characters of railroad wagon wheelsets in complex backgrounds. In this paper, ResNet [25] is used as the backbone network and combines the structure of FPN-PAN to construct a feature extraction network for multi-scale feature extraction.

**Figure 2:**
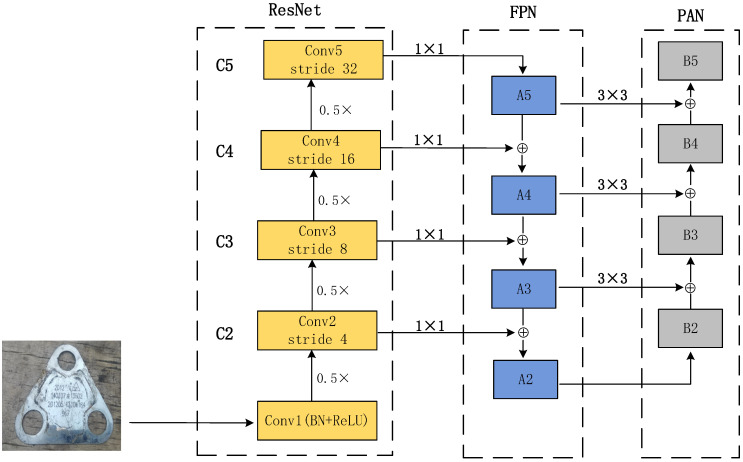
Feature extraction network architecture.

Deep feature maps have more semantic features, while shallow feature maps have more location information. FPN [26] conducts deep semantic features to shallow networks, thus enhancing semantic representations on multiple scales. At the same time, PAN [27] runs the shallow location information to the deep network to improve localization. Combining the two bidirectional feature pyramid modules enables better semantic and location data extraction; it enhances the multi-scale feature extraction capability of the whole network, and the specific network architecture is shown in Figure 2.

The image first passes through the ResNet50 network, extracting four hierarchical features (C2, C3, C4, C5). Four stages are used to form the feature pyramid, which is down-sampling concerning the image’s resolution to be 4, 8, 16, and 32. Starting from C5, the feature map is up-sampling by the nearest-neighbor method to obtain A5; the solution of C4 is adjusted by 1 × 1 convolution to obtain the exact resolution as A5; then, it is summed up line by line to obtain A4. This iterative method can realize the feature fusion of C3 and C2.

Each summed feature map (A3, A4, A5) is reduced in spatial size by a convolutional channel size of 3 × 3 and step size of 2, where A2 is the same as B2 and is not processed. After that, each element of the feature map A3 is firstly summed with the down-sampling map of B2 by lateral connection, and the fused feature mapping is processed through another 3 × 3 convolutional layer to generate the subsequent network B3, and this iterative process stops when approaching B5.

One copy of the feature information extracted from the feature extraction network is fed into the RPN, and one copy is provided to the ROI for processing, as shown in Figure 1.

#### 3.1.2. Region Proposal Network (RPN)

RPN generates anchor boxes in the feature image using a convolutional sliding window; then, it determines whether each anchor box is foreground or background, and it ultimately uses bounding box regression to adjust the anchor box to be closer to the ground truth box.

As a commonly used loss function in RPN, intersection over union (IOU) loss measures the similarity between the predicted bounding and ground truth boxes. It is the most widely used metric to measure the similarity between bounding boxes. Using IOU may result in cases where the predicted bounding box and ground truth box do not intersect, resulting in the IOU being zero.

**Figure 3:**
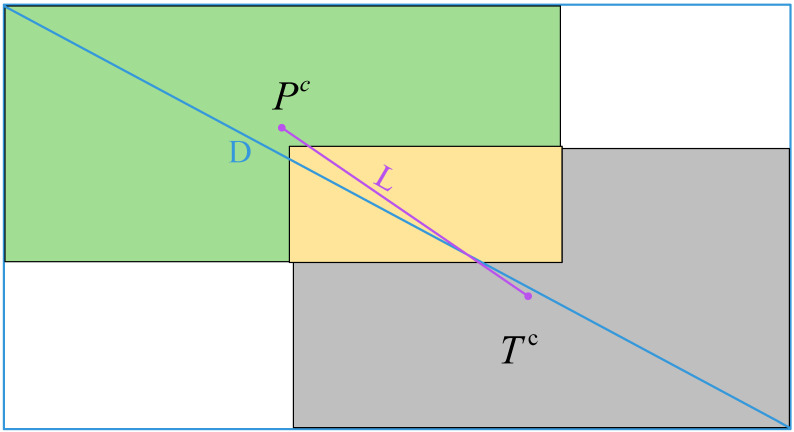
*DIOU* structure schematic.

DIOU [28] uses the Euclidean distance between the centers of the two bounding boxes and the distance between the two diagonal vertices of the smallest rectangular box for constraints, which not only takes into account the degree of overlap but also the differences between the positions and sizes, which improves the detection accuracy of the bounding box compared to IOU. The specific architecture is shown in Figure 3, where the green box is the annotation box, the gray box is the prediction box, and the yellow box is the intersection of the two; the blue border is the minimum bounding box.

When using *DIOU,* the penalty term is set to reduce the distance between the centroid of the prediction box and the ground truth box, and the specific formula is shown in Equation (1):

(1) $$R_{DIOU}=\frac{L^{2}(P^{c},T^{c})}{D^{2}}$$

The formula for *DIOU* is shown in Equation (2):

(2) $$DIOU=IOU-\frac{L^{2}(P^{c},T^{c})}{D^{2}}$$

*IOU* denotes the traditional intersection and concurrency ratio, $P^{c}$ indicates the center point of the prediction box, $T^{c}$ denotes the center point of the ground truth box, *L* denotes the Euclidean distance between the two centroids of the computation, and *D* denotes the diagonal length of the smallest closed region that can contains both the prediction frame and the target frame.

#### 3.1.3. Region of Interest (ROI)

Feature maps extracted by the feature extraction network and RPN are fed into ROI. After receiving the feature mapping, the ROI classification network uses the pooling operation to map the features of different sizes into small feature maps of the same size.

The horizontal and directional boxes are adjusted using parallel processing when processing candidate boxes in the input feature mapping. It is combined with bounding box regression and classification to regress and classify the position between the character and the background. Finally, three vectors are output: classification probability, horizontal bounding box regression offset, and directional bounding box regression offset.

Parameters (x,y,w,h) represent the ground truth of horizontal bounding boxes, the (x,y) coordinates are the horizontal boxes’ center point, and (w,h) are the horizontal boxes’ width and height. The parameters are constructed by adding an angle θ∈(−π/2,π/2) to describe the orientation information for oriented bounding boxes. As a multitasking problem, the parallel processing of horizontal and directional branches is more conducive to the convergence of the network, and the multitasking loss function is defined in Equation (3):

(3) $$L(p,\hat{p},q,\hat{q},r,\hat{r})=L_{cls}(\hat{p},\hat{p})+\lambda_{1}\sum_{i\in \left\{x,y,w,h\right\}}L_{reg}(q_{i},{\hat{q}}_{i})+\lambda_{2}\sum_{j\in \left\{x,y,w,h,\theta \right\}}L_{reg}(r_{j},{\hat{r}}_{j})$$

*p* and $\hat{p}$ represent the actual and predicted probabilities that the anchor point is a tilted character, $\lambda_{1}$ and $\lambda_{2}$ are the parameter weights balancing the two location branches, *q* and $\hat{q}$ represent the true and predicted probabilities of the horizontal bounding box, and *r* and $\hat{r}$ represent the actual and predicted probabilities of the rotated bounding box. $L_{cls}$ denotes the loss of bounding box classification correctness and $L_{reg}$ denotes the loss of regression branch classification correctness.

### 3.2. Tilted Character Correction Network

To facilitate text line detection in CRNN, detected candidate text boxes will be oriented horizontally but with the character reversal problem. Reversed character affects not only the efficiency of the network but also the output of the wrong information, which will produce a bad judgment on the message of the wheelset—affecting the safety of the operation of railroad wagons. Therefore, the character correction network is constructed to categorize reversed characters’ directions and correct the erroneous characters.

**Figure 4:**
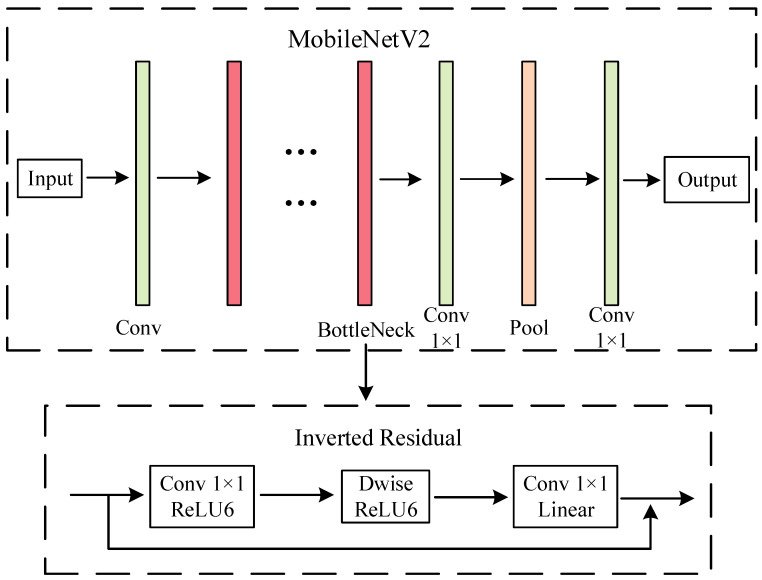
MobileNetV2 and inverted residual network architecture.

Based on the considerations of network model size and depth of network layers, the lightweight MobileNetV2 network model was chosen. The most prominent feature of MobileNetV2 is the inclusion of the inverted residual structure to reduce the amount of computation and the number of parameters. The MobileNetV2 and inverted residual design is shown in Figure 4, where Conv denotes the convolutional layer, Pool denotes the pooling layer, ReLU6 denotes the activation function, and Dwise denotes depthwise convolution.

**Figure 5:**
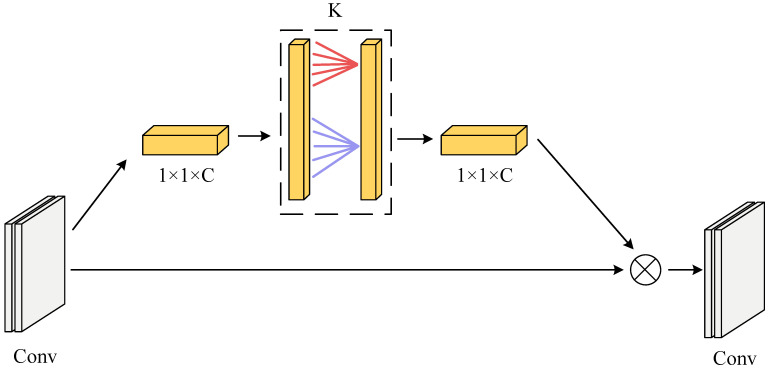
ECA attention module.

The features of the direction classification task for reversed characters are concentrated in a small area, and the feature differences are not noticeable. To improve the accuracy of character classification, the ECA [29] attention mechanism is added to enhance the extraction of character features; the structure is shown in Figure 5.

The ECA module uses one-dimensional convolution to capture local cross-channel interactional information through adaptive channel coverage *K*, which reduces the number of parameters while improving network performance. The channel coverage *K* indicates the local cross-channel interaction coverage and how many neighboring channels close to the channel participate in the attention prediction. The magnitude of *K* is determined by the adaptive Equation (4).

(4) $$K={\left|\frac{\log_{2}(C)}{\eta }-\frac{b}{\eta }\right|}_{odd}$$

*C* is the input feature channel’s dimension, ${\left|X\right|}_{odd}$ is the closest odd number to *x*, η and *b* is taken as 2 and 1. Because of the limitations of the linear mapping relationship in terms of specific relevant properties, the mapping relationship uses nonlinear mapping.

Adding the ECA attention mechanism to the inverted residual module of MobileNetV2 constitutes the MobileNetV2_ECA network. The 1 × 1 pointwise convolution features are fed into the ECA module as input information. The input feature information interacts with the announcement of the neighboring *K* channels after global average pooling. The interacted feature information is multiplied with the input features to generate the output features.

**Figure 6:**
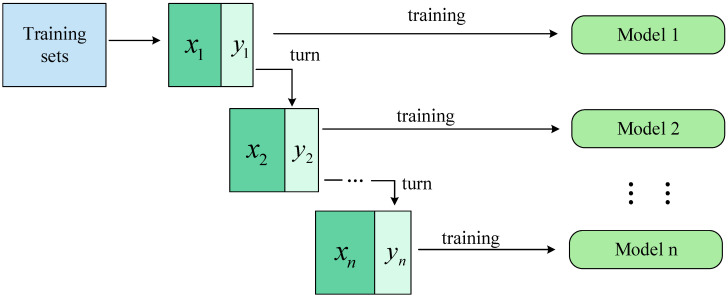
Structure of the Bagging algorithm.

To improve the accuracy of the tilted character correction network, the Mobile_ECA network is used as an individual learner, and the Bagging algorithm with parallel generative computing is used for training. The algorithm uses a random sampling operation with a put-back for individual learners; the diversity among individual learners is increased by the perturbation of the samples, as shown in Figure 6. Here, $x_{1},x_{2}\ldots xn$ represent the data collected when random sampling is performed, which is put back into the dataset after the training is completed; $y_{1},y_{2}\ldots y_{n}$ represent the data that are not collected when random sampling is performed; model represents the training model, which is MobileNetV2_ECA in this paper.

Considering the network structure parameters and the model’s size, this paper uses three MobileNetV2_ECA networks for training. The three output predictions are processed using the weighted voting method as shown in Equation (5):

(5) $$H(x)=C_{\arg\max q}\sum_{m=1}^{M}\omega_{m}h_{m}{}^{q}(x)$$

$h_{m}$ denotes the individual learner, $\omega_{m}$ means the weights of $h_{m}$, and the weight coefficients are often chosen to be normalized for processing and are constrained to be $\omega_{m}\geq 0$ and $\sum_{m=1}^{M}\omega_{m}=1$.

### 3.3. Character Recognition Network

In addition to numbers and letters, Chinese characters are also on the steel stamps produced for railroad wagon wheelsets. Therefore, the training of special Chinese characters will be added to the network training. The CRNN network is selected for character information recognition to recognize characters more accurately, and they are mainly composed of a convolutional, recurrent, and transcription layer.

The convolutional layer uses MobileNetV2_ECA to divide the input image into *M* different image patches while generating *M* feature vectors. The extracted *M*-corresponding feature vectors are transferred to the recurrent layer by convolution and pooling.

**Figure 7:**
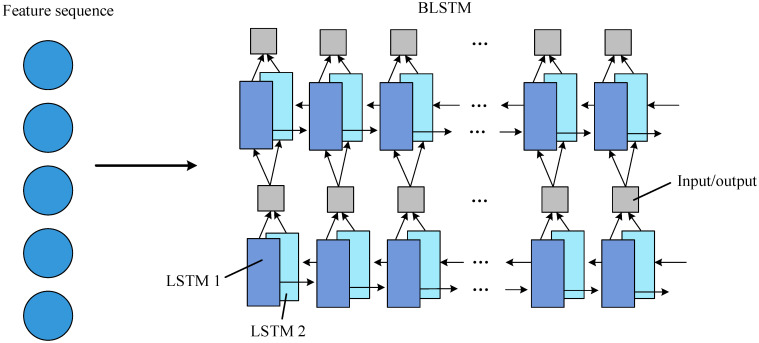
Recurrent layer network architecture.

The recurrent layer operates on text sequences of arbitrary length using BLSTM while capturing contextual information for more accurate recognition. As shown in Figure 7, each BLSTM [30] consists of LSTM1 and LSTM2, with one of the two LSTMs doing self-loop operations. Multiple BLSTMs can be stacked to realize the transition from feature information input to state sequence output.

The transcription layer uses CTC as a loss function to convert each frame prediction made by the recurrent layer into a sequence of tags and blanks, ultimately realizing a text sequence corresponding to the input image.

## 4. Experiments and Result

### 4.1. Experimental Configuration

To prove that the method is effective, the dataset for this experiment uses 1000 images from actual factories, which mainly contain pictures of multi-angle wheelsets’ character information. These 1000 images are divided into a training set and test set according to the ratio of 7:3, and the 700 images in the training set are expanded with data enhancement methods such as random rotation and Gaussian blurring, and finally, the data-enhanced dataset is labeled using the tool roLabelImg. Among them, the detection network contains the labeling information of the detection frame and angle, the correction network contains the labeling information of the detection frame, “positive” and “negative”, and the recognition network contains the labeling information of the detection frame, numbers, and letters. The experimental hardware environment is shown explicitly in Table 1, and the detection network, correction network, and recognition network training parameters are shown in Table 2.

In order to conduct better experiments on network testing, we use metrics such as Accuracy and mean average precision (mAP) for evaluation. The *Accuracy* and mAP calculation formulas are shown in Equations (6) and (7).

(6) $$Accuracy=\frac{TP+TN}{TP+TN+FP+FN}$$

*TP* denotes the case where the prediction is positive and the actual is positive; *FP* denotes the case where the prediction is positive and the actual is negative; *FN* denotes the case where the prediction is negative and the actual is positive; and *TN* denotes the case where the prediction is negative and the actual is negative.

(7) $$mAP=\frac{\sum_{i=1}^{K}AP_{i}}{K}$$

where K∈(2,3,4⋯), and the equation for *AP* is shown in Equation (8).

(8) $$AP=\sum_{m=1}^{n-1}\left(r_{i+1}-r_{i})P_{inter}(r_{i}+1\right)$$

where $r_{1},r_{2}\ldots r_{n}$ are the *Recall* values corresponding to the first interpolation at the first interpolation of the *Precision* interpolation segment in ascending order. The formulas for *Precision* and *Recall* are shown in Equations (9) and (10).

(9) $$Precision=\frac{TP}{TP+FP}$$

(10) $$Recall=\frac{TP}{TP+FN}$$

### 4.2. Experimental Analysis

Comparisons between different detection network architectures for the dataset are given in Table 3, which includes the results of the comparison experiments between the traditional template-matching character recognition method and the Faster RCNN network architecture as well as the results of the ablation experiments between the Faster RCNN network architecture and the improved method.

Table 3 shows that the detection speed and accuracy of the traditional template-matching method are much lower than that of the deep learning method. Adding the FPN-PAN module to the Faster RCNN network or using DIOU-Loss, respectively, both improve the original network’s accuracy but with a slight increase in detection time. Incorporating both modules into the Faster RCNN network resulted in a 2% increase in network accuracy and a 2% increase in mAP.

Table 4 shows the experimental accuracy results, number of parameters, and prediction time for MobileNetV2, MobileNetV2_ECA, and the three MobileNetv2_ECAs computed by the ensemble algorithm. It shows that after adding the ECA module, the accuracy of MobileNetV2 is improved without changing the number of parameters. After using the ensemble algorithm on MobileNetV2_ECA, the accuracy of the whole network is enhanced by 4% compared to the MobileNetV2 network, and there is only a 1.5 ms increase in the inference time.

Recognition network architecture CRNN uses MobileNetV2_ECA as the feature extraction network, the network model size is 13 Mb, the operation time is 16 ms, and the recognition accuracy of characters reaches 96.3% at the same time; the specific experimental data are shown in Table 5; the experiment shows that the recognition network architecture constructed in this paper is able to maintain a high accuracy rate for character recognition while ensuring a lightweight architecture.

**Figure 8:**
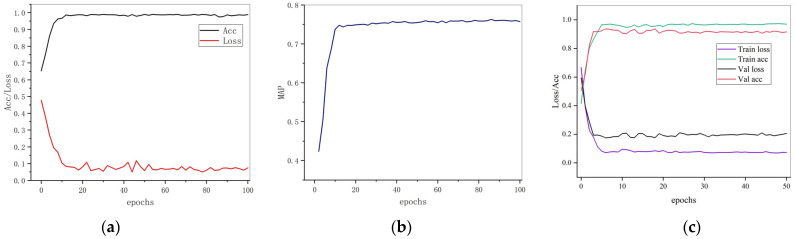
Training results of network. (**a**) shows the ACC and loss for detecting network training, (**b**) shows the mAP for detecting network training, (**c**) shows the Accuracy and loss for correction network.

The training changes of the accuracy and loss function of the whole detection network are shown in Figure 8a, and the changes in the mAP index of the entire detection network are shown in Figure 8b. The correction network training’s accuracy rate and loss function are shown in Figure 8c.

**Figure 9:**
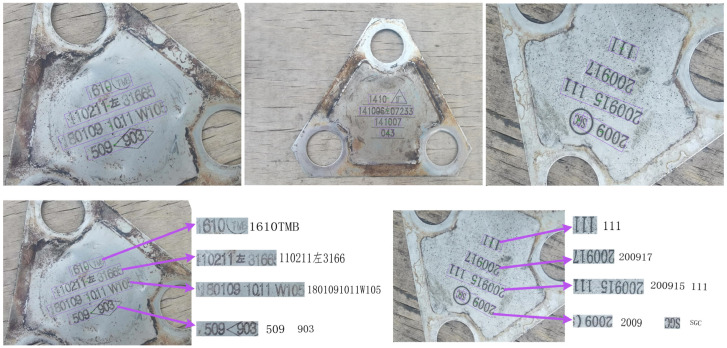
Overall effectiveness of network detection and recognition. The output forms of the characters in the figure are all pictures. There are a few Chinese characters, for example, “左” and “厂” represent the meaning of “left” and “factory” respectively.

Figure 9 is a diagram of the results of the overall method for detecting railroad wagon tilted wheelset characters, which includes the results of detecting and recognizing unflipped characters and detecting and recognizing flipped characters.

## 5. Conclusions

In this paper, a deep learning-based method for the tilted character recognition of railway wagon wheelsets is proposed, which mainly realizes the task of detecting and recognizing tilted characters in wagon wheelsets’ images. and the process consists of three parts: tilted character detection network, tilted character correction network, and tilted character recognition network. The tilted character detection network is improved based on the Faster RCNN model, which improves the speed of the detection network as well as enhances the regression accuracy and the recognition accuracy of the detection network so that the whole tilted character detection network can quickly and accurately detect the tilted character information in the wheelset image. The tilted character correction network combines MobileNetV2 with ECA. It adds integrated operations to improve the ability of the tilted character correction network to extract features across channels, the accuracy of orientation classification, and the speed of correction to realize the task of orientation classification and revision of the flipped characters removed by the detection network. The tilted character recognition network is based on CRNN and realizes the mission of accurately recognizing the corrected indeterminate length characters. Experiments conducted on tilted wagon wheelset characters demonstrate that the detection network achieves a mAP of 74.6%, and the accuracy of both the correction network and the recognition network exceeds 94%. However, the accuracy of the network needs to be improved when recognizing special symbols. In the future, by combining lightweight and convenient devices, it will be possible to realize an easier recognition of character information and lower computing costs.

## Figures and Tables

**Table 1:** Hardware environment configuration.

Configuration	Versions
CPU	12th Gen Intel (R) Core (TM) i5-12500H
RAM	16.0 GB
Operating system	Windows 11
Language	Python 3.10
Framework	Pytorch 1.11.0

**Table 2:** Network parameters.

Net	Training Sets (Base)	Validation Sets (Base)	Batch Size	Learning Rate
Detection	700	300	50	0.0001
Correction	700	300	50	0.0001
Discriminate	700	300	50	0.0001

**Table 3:** Comparison of detection network architectures.

Model	Accuracy (%)	mAP (%)	Inference Time (ms)
Template matching	82.95	-	203
Faster (ResNet)	92.33	72.6	30.6
Faster + FPN-PAN	93.21	73.4	32.7
Faster + DIOU	93.19	73.1	32.3
Faster + FPN-PAN + DIOU	94.69	74.6	34.6

**Table 4:** Comparison of MobileNetV2, MobileNetV2_ECA, and Ensemble models.

Model	Accuracy (%)	Parameters (M)	Inference Time (ms)
MobileNetV2	90.13	3.5	5.7
MobileNetV2_ECA	92.34	3.5	6.5
Ensemble	94.62	11.6	8.2

**Table 5:** Discriminate network model.

Model	Accuracy (%)	Model Size (Mb)	Inference Time (ms)
CRNN (ResNet)	94.73	27	23.11
CRNN (MobileNetV2)	94.12	11	15.89
CRNN (MobileNetV2_ECA)	96.36	13	16.13

## Data Availability

Data available on request due to restrictions, e.g., privacy or ethics.
